# Insulin and α-Tocopherol Enhance the Protective Effect of Each Other on Brain Cortical Neurons under Oxidative Stress Conditions and in Rat Two-Vessel Forebrain Ischemia/Reperfusion Injury

**DOI:** 10.3390/ijms222111768

**Published:** 2021-10-29

**Authors:** Irina O. Zakharova, Liubov V. Bayunova, Inna I. Zorina, Tatiana V. Sokolova, Alexander O. Shpakov, Natalia F. Avrova

**Affiliations:** I.M. Sechenov Institute of Evolutionary Physiology and Biochemistry, Russian Academy of Sciences, 194223 St. Petersburg, Russia; zakhar@iephb.ru (I.O.Z.); bayunova@iephb.ru (L.V.B.); carry111@yandex.ru (I.I.Z.); sokolova@iephb.ru (T.V.S.); alex_shpakov@list.ru (A.O.S.)

**Keywords:** insulin, α-tocopherol, protection, cortical neurons, oxidative stress, brain cortex, forebrain ischemia, reperfusion

## Abstract

Clinical trials show that insulin administered intranasally is a promising drug to treat neurodegenerative diseases, but at high doses its use may result in cerebral insulin resistance. Identifying compounds which could enhance the protective effects of insulin, may be helpful to reduce its effective dose. Our aim was thus to study the efficiency of combined use of insulin and α-tocopherol (α-T) to increase the viability of cultured cortical neurons under oxidative stress conditions and to normalize the metabolic disturbances caused by free radical reaction activation in brain cortex of rats with two-vessel forebrain ischemia/reperfusion injury. Immunoblotting, flow cytometry, colorimetric, and fluorometric techniques were used. α-T enhanced the protective and antioxidative effects of insulin on neurons in oxidative stress, their effects were additive. At the late stages of oxidative stress, the combined action of insulin and α-T increased Akt-kinase activity, inactivated GSK-3beta and normalized ERK1/2 activity in cortical neurons, it was more effective than either drug action. In the brain cortex, ischemia/reperfusion increased the lipid peroxidation product content and caused Na^+^,K^+^-ATPase oxidative inactivation. Co-administration of insulin (intranasally, 0.25 IU/rat) and α-T (orally, 50 mg/kg) led to a more pronounced normalization of the levels of Schiff bases, conjugated dienes and trienes and Na^+^,K^+^-ATPase activity than administration of each drug alone. Thus, α-T enhances the protective effects of insulin on cultured cortical neurons in oxidative stress and in the brain cortex of rats with cerebral ischemia/reperfusion injury.

## 1. Introduction

Co-administration of various neuroprotective agents that are capable of enhancing each other’s protective effects by modulating intracellular signaling pathways can significantly increase the ability of organisms to prevent the development of neuropathies in neurodegenerative, ischemic, or diabetic brain lesions. This is especially important in the case of insulin, since long-term exposure to high concentrations of this hormone leads to insulin resistance, it was shown to be characteristic for type 2 diabetes mellitus, Alzheimer’s and Parkinson’s diseases and other endocrine pathologies [1,2,3]. In accordance with the above, an urgent task for neurochemistry and neuroendocrinology is the search for endogenous regulators and natural substances, which could make it possible to reduce the pharmacological doses of insulin without causing a decrease in its neuroprotective effect.

Insulin is currently one of the most promising neuroprotective agents that are widely used in clinical trials. In pathological conditions such as type 2 diabetes mellitus, metabolic syndrome, Alzheimer’s and Parkinson’s diseases, the insulin level is increased markedly in blood, while in the brain, on the contrary, it is markedly diminished, which leads to disruption of insulin signaling in the hypothalamus and other regions of the brain [4,5,6,7]. Decreased levels of insulin in the brain and central insulin resistance lead to impaired metabolism and functional activity of neurons, as well as to the changes in hypothalamic regulation of carbohydrate and lipid metabolism, food intake and endocrine functions [8,9,10].

One of the most promising ways to correct insulin deficiency in the brain is its intranasal administration, since in this case the hormone enters the brain directly with the participation of cells of the olfactory and trigeminal nerves, bypassing the blood-brain barrier [11,12]. The neuroprotective effect of intranasally administered insulin is supported by numerous studies on the prevention of neurodegenerative brain damage in experimental animals [1,13,14]. The use of intranasally administered insulin for the treatment of patients with Alzheimer’s disease and mild cognitive deficits leads to a significant improvement in their cognitive functions (see, for example, [15,16,17,18,19]). The clinical trials of intranasally administered insulin as a drug for the treatment of Parkinson’s disease are also in progress [20].

There is reason to believe that intranasally administered insulin can have a neuroprotective effect in the case of cerebral ischemia, but information on this is extremely scarce. There are only our previous works on the ability of intranasally administered insulin to normalize metabolism and redox processes in the ischemic and reperfused brain [21,22]. Recently, a protective effect of intracerebroventricularly administered insulin on the viability of brain neurons in gerbils with global ischemia followed by reperfusion has been shown [23]. Ischemic brain damage is much more pronounced in rats with diabetes mellitus than in healthy animals, as a result of which systemic treatment of diabetic animals with insulin weakened the severity of ischemic lesions [24,25]. There are numerous studies, the authors of which have shown the protective effect of intranasally administered insulin-like growth factor-1 (IGF-1) in animals with focal and global ischemia (see, for example [26,27,28]). At the same time, in their review article, Lioutas and coauthors [29] noted that intranasally administered insulin is a more promising neuroprotector than IGF-1 as a drug for preventing severe consequences of ischemic brain damage, including acute ischemic stroke.

Among the compounds that may enhance the neuroprotective effect of insulin, α-tocopherol (α-T), the main and most active component of vitamin E, one of the natural components of various brain cells, is of particular interest (see, for example, [30,31,32]). In one of the recent mini-reviews [32] it is emphasized that targeting insulin resistance may be a breakthrough strategy to treat Alzheimer’s disease. It is of interest that vitamin E and C are considered to be able to decrease insulin resistance due to their antioxidant effects [32]. High doses of α-T or vitamin E are usually used in clinical trials to improve cognitive function in Alzheimer’s patients. It is important to note that peripheral α-T levels in patients with Alzheimer’s disease and mild cognitive impairment were lower than in healthy people, with the difference being quite significant [33]. These data suggest a relationship between vitamin E and α-T content in diet and the risk of developing Alzheimer’s disease and age-related cognitive deficit.

The aim of our work is to test the hypothesis that insulin and α-T can significantly enhance the neuroprotective effects of each other in the in vitro and in vivo experiments. We studied the neuroprotective, antiapoptotic and antioxidant effects of insulin and α-T on brain cortical neurons under conditions of oxidative stress, comparing the effects of individual drugs with the effects of their combined use. To elucidate the possible molecular mechanism of the additive neuroprotective action of insulin and α-T, the individual and joint effects of these neuroprotectors on the activity of protein kinase B (Akt), glycogen synthase kinase-3beta (GSK-3beta) and extracellular signal-regulated kinase 1/2 (ERK1/2) were studied. In the in vivo experiments, the ability of intranasally administered insulin and orally administered α-T to prevent or reduce the increased accumulation of different lipid peroxidation (LPO) products as well as oxidative inactivation of Na^+^,K^+^-ATPase in the cerebral cortex of rats with two-vessel forebrain ischemia and subsequent reperfusion was investigated. In this case, a comparative study of monotherapy and combined therapy with these drugs was carried out. Our data indicate that insulin and α-T are able to enhance the protective effects of each other on cortical neurons under conditions of oxidative stress and on the cells of the cerebral cortex of rats with ischemia/reperfusion injury.

## 2. Results

### 2.1. Additivity of Protective Effects of Insulin and α-Tocopherol on Viability of Rat Brain Cortical Neurons Exposed to Hydrogen Peroxide

**Figure 1:**
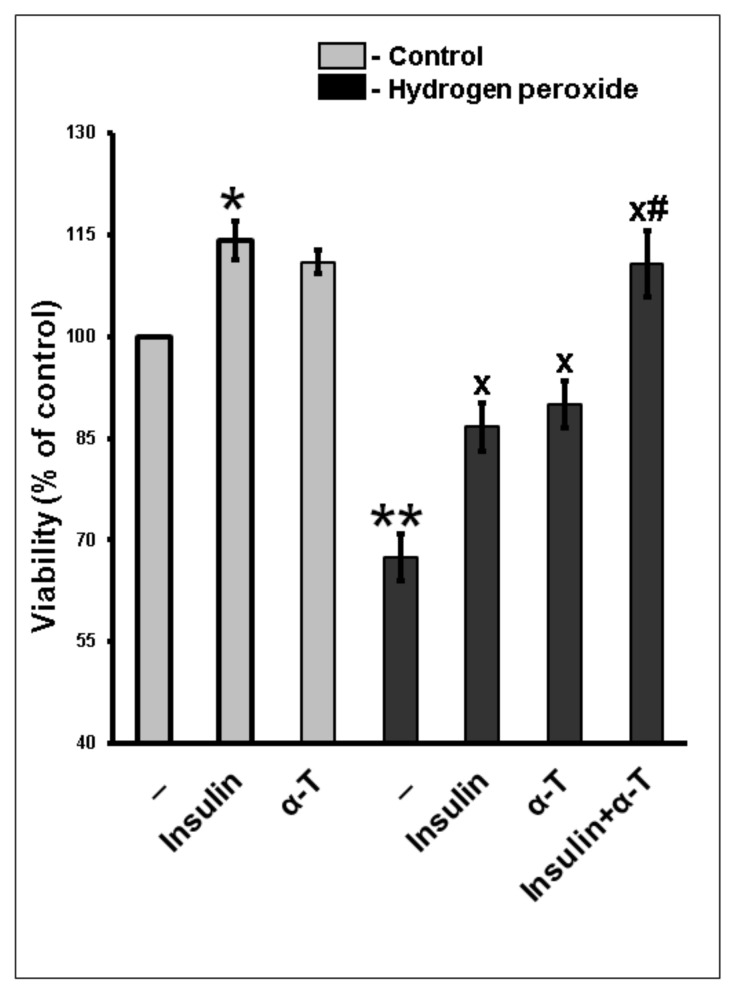
The additive protective effect of insulin and α-tocopherol (α-T) on the viability of rat brain cortical neurons exposed to hydrogen peroxide. The data of 5–7 experiments are presented as the means ± SEM. The neuronal viability was determined using 3-(4,5-dimethy-2-thiazolyl)-2,5-diphenyl-2*H*-tetrazolium bromide (MTT) method. Abbreviations used: α-tocopherol (α-T). Cortical neurons were pre-incubated for 1 h with 1 μM insulin or for 18 h with 50 μM α-T or with both compounds or without them. Then the neurons were exposed to 100 μM hydrogen peroxide for 6 h. The differences are significant according to one-way analysis of variance (ANOVA) followed by Tukey’s test for multiple comparisons as compared: *—to control, x—to the effect of hydrogen peroxide alone, #—to the effect of either insulin, or α-T alone, * *p* < 0.05, ** *p* < 0.01. in all other cases *p* < 0.01.

The treatment of cultured brain cortical neurons with 1 μM insulin and 50 μM α-T led to an increase in their viability under the conditions of oxidative stress induced by 100 μM hydrogen peroxide. The protective effects of insulin and α-T on neurons were found to be additive. The viability of neurons in the presence of both protectors was significantly higher than their viability in the presence of either insulin or α-T alone (Figure 1).

Thus, the data obtained provide evidence that insulin and α-T enhance the protective effect of each other on brain cortical neurons under oxidative stress conditions, increasing the viability of these cells.

### 2.2. Antiapoptotic Effect of Insulin Plus α-T on Rat Brain Cortical Neurons Exposed to Hydrogen Peroxide Is Higher than the Effects of Each Drug

**Figure 2:**
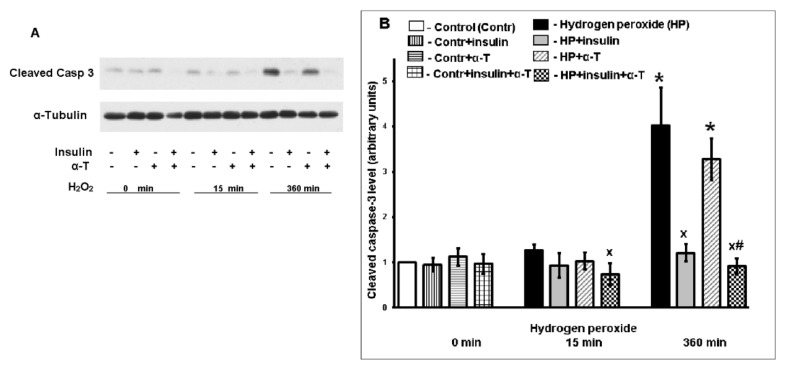
The antiapoptotic effect of insulin plus α-T on rat brain cortical neurons exposed to hydrogen peroxide is more pronounced than the effect of each drug. (**A**) immunoblots of cleaved caspase-3 are shown. (**B**) the data of 10 experiments are presented as the means ± SEM. Abbreviations used: Casp3–caspase-3. Cortical neurons were pre-incubated for 1 h with 1 μM insulin or for 18 h with 50 μM α-T or with both drugs or without them. Then the neurons were exposed to 100 μM hydrogen peroxide for 0, 15 min and 6 h. The differences are significant according to Student’s paired *t*-test as compared: *—to control, x—to the effect of hydrogen peroxide alone, #—to the effect of either insulin or α-T alone, *p* < 0.01 in all cases.

Apoptosis and necrosis are the most common neuronal death pathways under oxidative stress conditions. In order to show that apoptotic death occurred as a result of cortical neuron exposure to 100 μM hydrogen peroxide, the level of 17–19 kDa fragment of caspase-3 was analyzed. Its accumulation shows the cleavage and activation of this enzyme. In brain cortical neurons, the pronounced increase of cleaved caspase-3 level and hence of caspase-3 activation was observed 6 h after the initiation of oxidative stress by 100 μM hydrogen peroxide (Figure 2A,B). Pre-incubation with 1 μM insulin decreased the level of cleaved caspase-3, while the effect of 50 μM α-T was not observed. In the case of pre-incubation with both insulin and α-T, the decrease of cleaved caspase-3 level (and hence of neuronal apoptotic death) was more pronounced than in the case of pre-incubation with each of the protectors 6 h after application of hydrogen peroxide to the neurons (*p* < 0.01 by paired Student’s *t*-test). Even 15 min after the addition of pro-oxidant to cortical neurons, their pre-incubation with insulin plus α-T resulted in a significant decrease in cleaved caspase-3 level and hence of neuronal apoptotic death, while the addition of insulin or α-T alone had no effect on the neuronal apoptotic death (Figure 2B).

### 2.3. Antioxidative Effect of Insulin Plus α-T on Rat Brain Cortical Neurons Exposed to Hydrogen Peroxide Is Higher than the Effects of Each Drug

**Figure 3:**
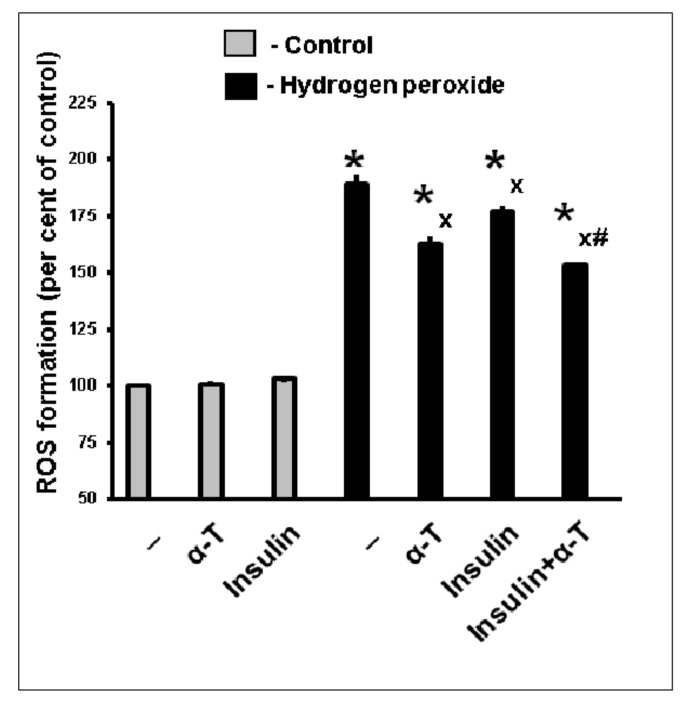
The pre-incubation with insulin or α-T or with both drugs diminishes the formation of ROS in brain cortical neurons exposed to hydrogen peroxide. The data of one typical experiment from five experiments made are given as the means ± SEM. Cortical neurons were pre-incubated for 1 h with 1 μM insulin or for 18 h with 50 μM α-T or with both drugs or without them. Then the neurons were exposed to 100 μM hydrogen peroxide for 1 h. The differences are significant according to Student’s *t*-test as compared to: *—control, x—the effect of hydrogen peroxide alone, #—the effect of either insulin or α-T alone, *p* < 0.05 in all cases.

Pre-incubation of brain cortical neurons with 50 μM α-T or 1 μM insulin diminished the formation of reactive oxygen species (ROS) in these cells induced by hydrogen peroxide exposition. The results of a typical experiment are shown in Figure 3. The use of insulin plus α-T was more effective than insulin or α-T alone (*p* < 0.05).

### 2.4. Effect of Insulin and α-T on Activity of Protein Kinase B (Akt) in Control and Hydrogen Peroxide-Exposed Brain Cortical Neurons

To assess the mechanisms of α-T-enhancing effect on insulin-induced neuroprotection in cortical neurons, we compared the combined and separate effects of these drugs on the activity of the protein kinases Akt, GSK-3beta and ERK1/2. The activity of Akt was assessed using Western blotting, for which the level of Ser^473^- and Thr^308^-phosphorylated forms of Akt kinase was measured using monoclonal antibodies specific for pAkt (Ser^473^) and pAkt (Thr^308^), respectively. The data obtained showed that insulin and hydrogen peroxide increased the phosphorylation of both residues. Nevertheless, the intensity of Thr^308^-phosphorylation was less pronounced than the intensity of Ser^473^-phosphorylation, so further experiments were carried out mainly using antibodies against pAkt (Ser^473^).

**Figure 4:**
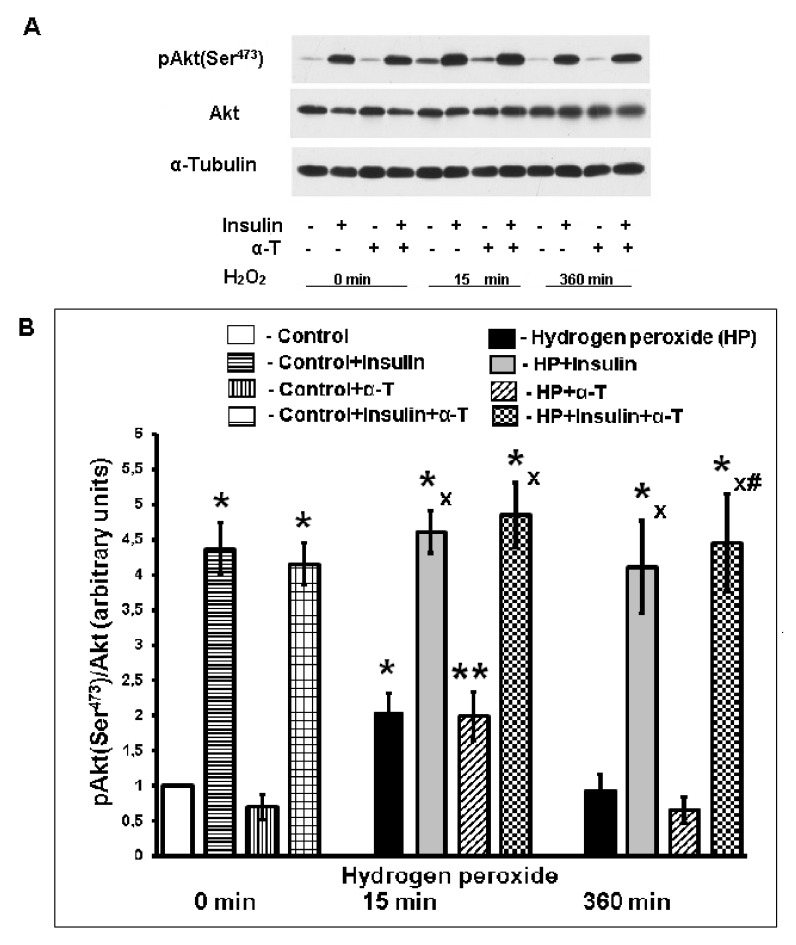
The effect of hydrogen peroxide, insulin and α-T on the activity of Akt (pAkt (Ser^473^)/Akt) in brain cortical neurons. (**A**) immunoblots of pAkt (Ser^473^) and total Akt are shown; (**B**) the data of 6 experiments are presented as the means ± SEM. The brain cortical neurons were pre-incubated for 1 h with 1 μM insulin or for 18 h with 50 μM α-T or with both drugs or without them. Then the neurons were exposed to 100 μM hydrogen peroxide for 0, 15 and 360 min. The differences are significant according to Student’s paired *t*-test as compared: * and **—to the control, * *p* < 0.02, ** *p* < 0.05; x—to the effect of hydrogen peroxide alone, *p* < 0.01, #—to the effect of insulin or α-T alone, *p* < 0.01.

Hydrogen peroxide increased Akt activity 15 min after its addition to neurons but had no effect on Akt activity 6 h after its addition to these cells. At the same time, pre-incubation with insulin led to activation of Akt kinase both in control neurons and in neurons exposed to hydrogen peroxide for 15 min and 6 h. It was shown that 1 μM insulin increased the basal Akt activity more than four times. The effect of insulin plus α-T was significantly higher than the effect of each of the drugs 6 h after pro-oxidant addition to the samples (*p* < 0.01 by paired Student’s *t*-test) (Figure 4). It is of importance that insulin alone and to a greater extent in combination with α-T could still increase the Akt activity when the Akt activating effect of hydrogen peroxide had already disappeared (Figure 4).

### 2.5. Effect of Insulin and α-T on pGSK-3beta (Ser^9^) Level in Control and Exposed to Hydrogen Peroxide Brain Cortical Neurons

**Figure 5:**
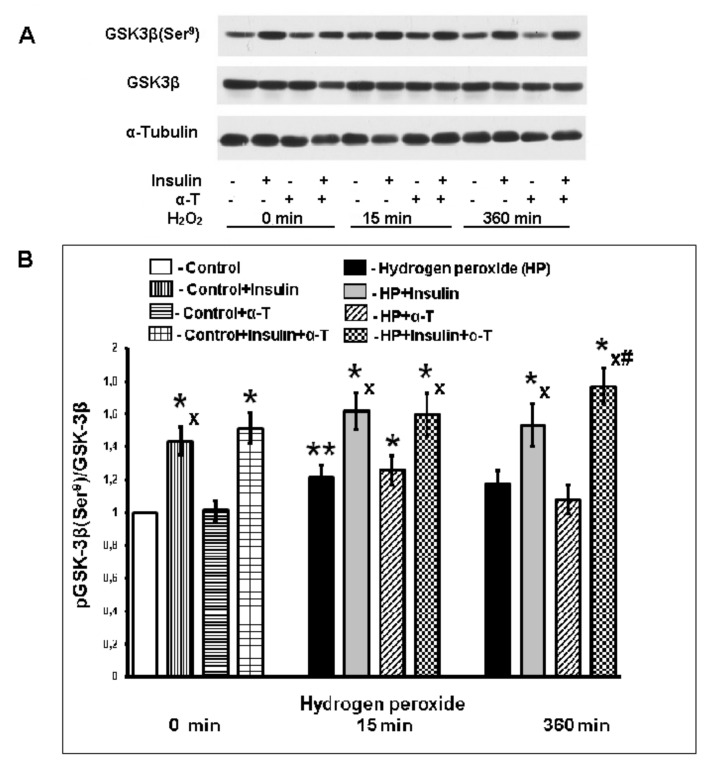
The effect of hydrogen peroxide, insulin and α-T on the activity of protein kinase GSK-3beta [pGSK-3beta (Ser^9^)/GSK-3beta] in brain cortical neurons. (**A**) immunoblots of pGSK-3beta (Ser^9^) and total GSK-3beta are shown; (**B**) the data of 10 experiments are presented as the means ± SEM. The brain cortical neurons were pre-incubated for 1 h with 1 μM insulin or for 18 h with 50 μM α-T or with both drugs or without them. Then the neurons were exposed to 100 μM hydrogen peroxide for 0, 15 and 360 min. The differences are significant according to Student’s paired *t*-test as compared: * and **—to the control, * *p* < 0.02, ** *p* < 0.05; x—to the effect of hydrogen peroxide alone, *p* < 0.01, #—to the effect of insulin or α-T alone, *p* < 0.01.

Akt activation by the drugs or pro-oxidants may lead to phosphorylation of GSK-3beta at the Ser^9^ and, as a result, inactivates this enzyme. 15 min after its addition to brain cortical neurons, hydrogen peroxide induced the activation of Akt kinase and increased the ratio pGSK-3beta (Ser^9^)/GSK-3beta. But had no effect (*p* > 0.05) on these enzymes 6 h after the induction of oxidative stress (Figure 4 and Figure 5), The effect of insulin on both the pGSK-3beta level, and the Akt activity was much more pronounced and prolonged as compared to the effect of hydrogen peroxide (Figure 4 and Figure 5). Insulin increased the ratio pGSK-3beta (Ser^9^)/GSK-3beta in control neurons from 1.0 to 1.43 ± 0.09 (*p* < 0.01). Under the conditions of oxidative stress, its ability to increase this ratio and hence to inactivate GSK-3beta was significant and pronounced (*n* = 10, *p* < 0.01, paired Student’s *t*-test, Figure 5B). In contrast to insulin, the ability of α-T to inactivate GSK-3beta in control neurons and in neurons 15 min and 6 h after the application of pro-oxidant was not detected.

α-T was found to enhance the effect of insulin 6 h after the induction of oxidative stress by hydrogen peroxide, the increase in pGSK-3beta (Ser^9^) levels in cortical neurons was significantly higher when cells were incubated with insulin plus α-T, as compared to incubation with insulin or α-T alone (*n* = 10, *p* < 0.01, paired Student’s *t*-test). It means that insulin is able to inactivate GSK-3beta in the course of oxidative stress development, while alpha-T is able to enhance its effect at late stages of oxidative stress development. The activation of GSK-3beta negatively affects the function of mitochondria in cells, including neurons, which leads to a decrease in the mitochondrial membrane potential and the opening of the mitochondrial permeability transition pore (mPTP). Thus, the above-mentioned inhibitory effects of insulin on GSK-3beta activity and the ability of α-T to enhance this effect appear to be of importance to provide their protective effects.

### 2.6. Effect of Insulin and α-T on ERK1/2 Activity (pERK1/2/ERK1/2 Ratio) in Control and Hydrogen Peroxide-Exposed Brain Cortical Neurons

The modulation of ERK1/2 activity may be of importance for the ability of insulin and α-T to increase the viability of cortical neurons. It was shown previously that short activation of ERK1/2 by the protectors increased the viability of nerve cells [34,35,36,37], but activation of ERK1/2 for long time promoted nerve cells death [36,37,38].

**Figure 6:**
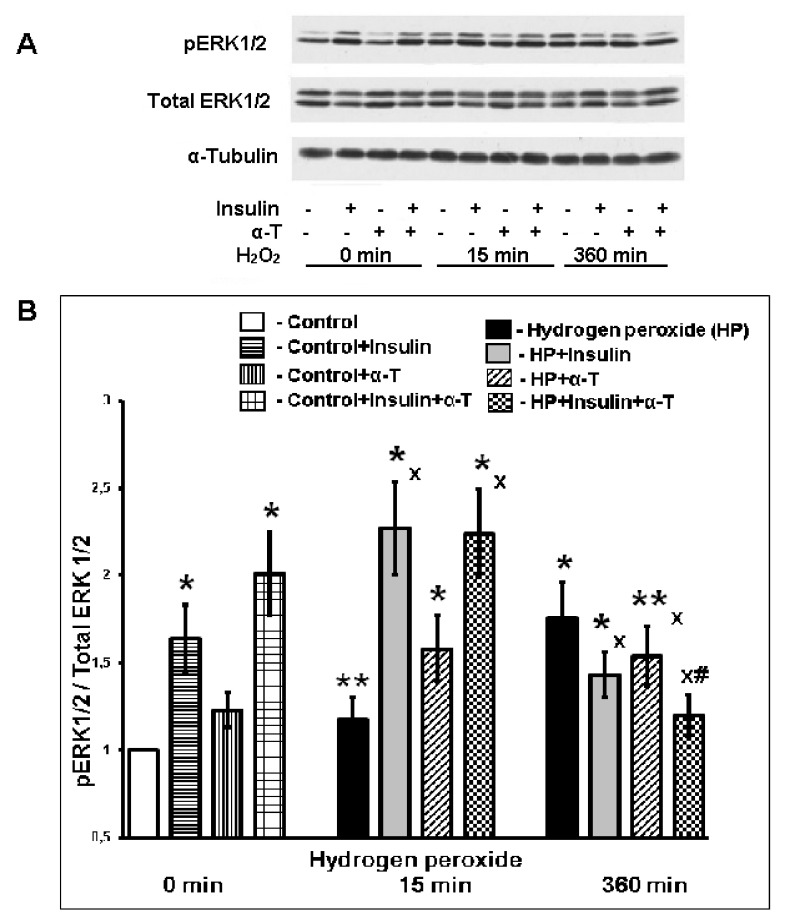
The effect of hydrogen peroxide, insulin and α-T on the activity of ERK1/2 (pERK1/2/ERK1/2 ratio) in brain cortical neurons. (**A**)—immunoblots of pERK1/2 and total ERK1/2 are shown; (**B**)—the data of 6 experiments are presented as the means ± SEM. The brain cortical neurons were pre-incubated for 1 h with 1 μM insulin or for 18 h with 50 μM α-T, with both drugs or without them. Then the neurons were exposed to 100 μM hydrogen peroxide for 0, 15 and 360 min. The differences are significant according to Student’s paired *t*-test as compared: * and **—to the control, * *p* < 0.02, ** *p* < 0.05; x—to the effect of hydrogen peroxide alone, *p* < 0.01, #—to the effect of insulin or α-T alone, *p* < 0.01.

In the present study, hydrogen peroxide increased the ERK1/2 phosphorylation 15 min after its application to the neurons (Figure 6). Insulin increased the basal ERK1/2 activity and its activity 15 min after the application of hydrogen peroxide (Figure 6). At this point, α-T effect was not detected. It was previously shown [39] that α-T increased the ERK1/2 activity only 5 min after hydrogen peroxide addition to cortical neurons. Hydrogen peroxide caused a pronounced increase of ERK1/2 activity at late stages of oxidative stress development—6 h after its application to the neurons (Figure 6). At this point, insulin and α-T decreased the ERK1/2 activity (*n* = 10, *p* < 0.05 by paired Student’s *t*-test). A decrease in the ERK1/2 activity was more pronounced in the case of insulin and α-T combination, and the difference between the effect of insulin plus α-T and the effects of insulin or α-T alone was significant (*n* = 10, *p* < 0.02 by paired Student’s *t*-test).

### 2.7. Effect of Insulin and α-T on Mitochondrial Membrane Potential in Control and Hydrogen Peroxide-Exposed Brain Cortical Neurons

The exposure of brain cortical neurons to hydrogen peroxide markedly diminished the mitochondrial membrane potential [∆ψ(m)] in brain cortical neurons—from 70.4 ± 2.09 arbitrary units in control neurons to 55.9 ± 4.64 arbitrary units in hydrogen peroxide-treated neurons (*p* < 0.05) (Table 1). It was shown by flow cytometry that the pre-incubation of neurons with 50 μM α-T or 1 μM insulin or with both drugs for 20 h resulted in the marked and significant (*p* < 0.02) increase of mitochondrial membrane potential of brain cortical neurons (see Table 1). The increase of ∆ψ(m) was more pronounced if neurons were pre-incubated with insulin+ α-T than in the case of their pre-incubation with one of these protectors, the differences being significant (*p* < 0.01) (Table 1). Thus, α-T was found to enhance the ability of insulin to increase ∆ψ(m) in neurons which was markedly diminished under conditions of oxidative stress.

**Figure 7:**
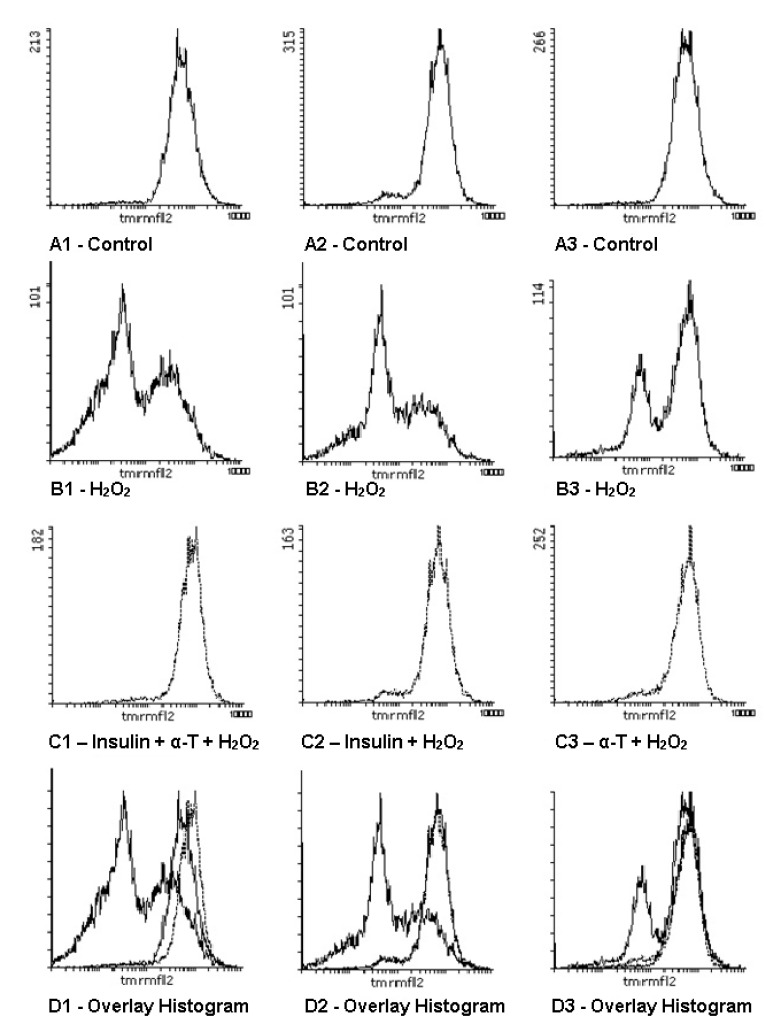
The effect of hydrogen peroxide, insulin, α-T and insulin plus α-T on the mitochondrial membrane potential in cortical neurons (histograms obtained by flow cytometry). Cortical neurons were pre-incubated with 1 μM insulin, 50 μM α-T or with both compounds. Then neurons were exposed to 100 μM hydrogen peroxide for 1 h. A1, A2, A3—controls, B1, B2, B3—the effect of hydrogen peroxide alone, C1, C2, C3—the effect of hydrogen peroxide after pre-incubation of neurons with: C1—1 μM insulin and 50 μM α-T, C2—1 μM insulin, C3—50 μM α-T, D1, D2, D3—the overlay of the histograms presented in A, B and C.

The histograms showing the control samples and the effect of hydrogen peroxide on cortical neurons in the absence of the protectors and after the pre-incubation with 1 μM insulin or with 50 μM α-T or with both compounds are given in Figure 7.

### 2.8. The Combination of Intranasally Administered Insulin Together with Orally Administered α-T Diminished the Accumulation of Schiff Bases in the Brain Cortex of Rats with Ischemia/Reperfusion to Higher Extent than Monotherapy with These Drugs

We studied the ability of insulin, α-T and their combinations to prevent or reduce metabolic disorders caused by the activation of free radical reactions in the cerebral cortex of rats with two-vessel forebrain ischemia and subsequent reperfusion. Two-vessel forebrain ischemia together with hypotension and subsequent reperfusion, led to a pronounced and significant increase in LPO products such as Schiff bases and conjugated dienes and trienes, and partially inactivated Na^+^,K^+^-ATPase in the cerebral cortex of rats. The ability of intranasally administered insulin and orally administered α-T to normalize the accumulation of lipid peroxidation products (LPO) and prevent oxidative inactivation of Na^+^,K^+^-ATPase in the cerebral cortex during ischemia and reperfusion was investigated.

**Figure 8:**
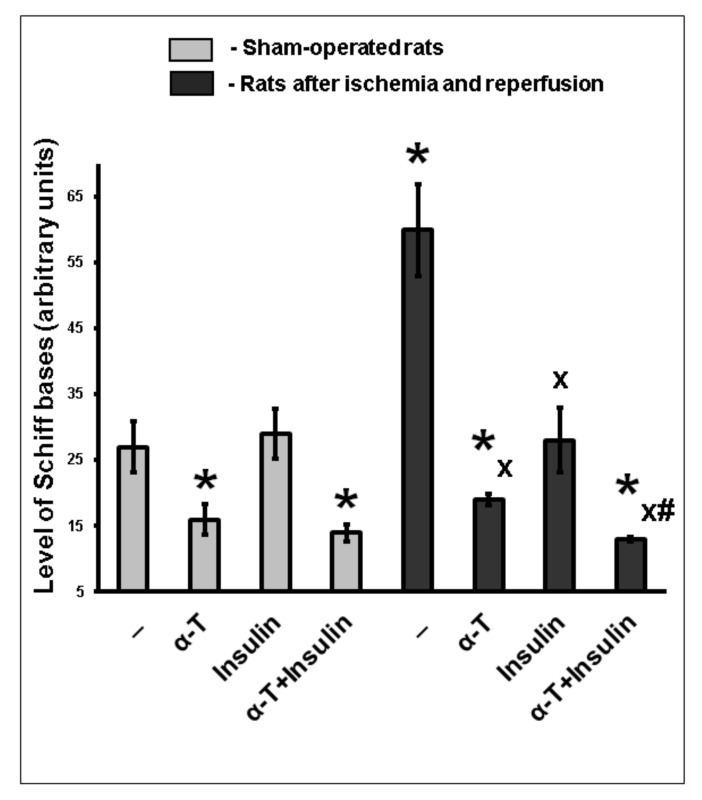
The effect of intranasally administered insulin and orally administered α-T on the content of Schiff bases in the brain cortex of rats after two-vessel forebrain ischemia followed by reperfusion. The data of 5–7 experiments are presented as the means ± SEM. The two-vessel ischemia was induced by ligation of carotid arteries for 20 min and hypotension, it was followed by reperfusion for 1 h. Insulin was administered intranasally at a dose of 0.25 IU/rat, while α-T was given orally at a dose of 50 mg/kg. The differences are significant according to Student’s *t*-test as compared: *—to the control, x—to the effect of ischemia and reperfusion, #—to the effect of insulin and α-T alone, *p* < 0.01 in all cases.

The content of Schiff bases increased more than two times in the brain cortex of rats with ischemia/reperfusion injury (Figure 8). The intranasally administered insulin (0.25 IU/rat) or orally administered α-T (50 mg/kg) decreased the content of Schiff bases (*p* < 0.01) (Figure 8). The administration of insulin plus α-T to rats decreased the level of Schiff bases in brain cortex to a higher extent than administration of insulin or α-T alone (*p* < 0.01) (Figure 8).

### 2.9. The Combination of Intranasally Administered Insulin Together with Orally Administered α-T Normalizes the Level of Conjugated Dienes and Trienes in the Brain Cortex of Rats with Ischemia/Reperfusion to a Greater Extent than Monotherapy with These Drugs

**Figure 9:**
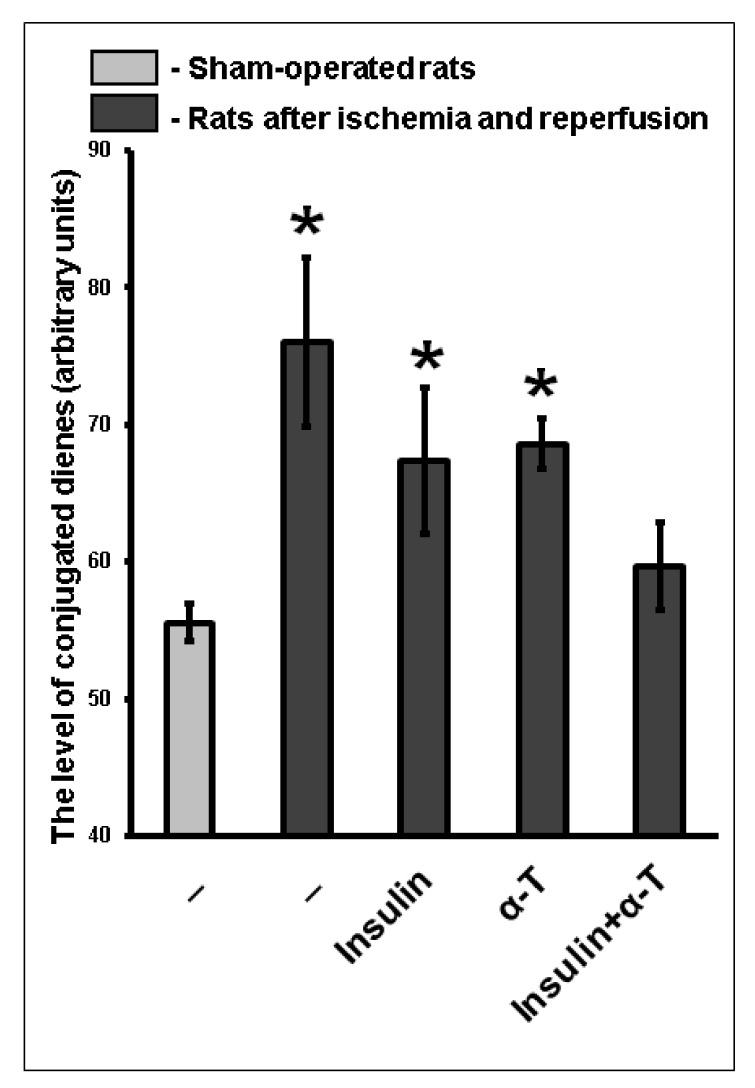
The effect of intranasally administered insulin and orally administered α-T on the level of conjugated dienes in the brain cortex of rats with two-vessel forebrain ischemia followed by reperfusion. The data of 5–7 experiments are shown as the means ± SEM. The two-vessel forebrain ischemia was induced by ligation of carotid arteries for 20 min and hypotension followed by reperfusion for 1 h. Insulin was administered intranasally at a dose of 0.25 IU/rat, while α-T was given orally at a dose of 50 mg/kg. *—The differences are significant according to Student’s *t*-test as compared to control values in the brain cortex of sham-operated rats, *p* < 0.01.

The level of conjugated dienes was markedly higher (by 36.8%) in the brain cortex of rats after ischemia and reperfusion as compared to sham-operated rats (*p* < 0.01). In the brain cortex of ischemic and reperfused rats which received either insulin (intranasally, 0.25 IU/rat) or α-T (orally, 50 mg/kg), the level of conjugated dienes was higher by 21.3% and 23.1%, respectively, as compared to sham-operated rats (*p* < 0.01) (Figure 9). At the same time, if both insulin and α-T were administered to rats with ischemic and reperfused forebrain in the above-mentioned doses, the level of conjugated dienes was not different from that in brain cortex of sham-operated rats (Figure 9). The additive effect of insulin and α-T was observed, as the total effect of both protectors administered to ischemic and reperfused rats was approximately equal to the sum of insulin ad α-T effects (Figure 9).

**Figure 10:**
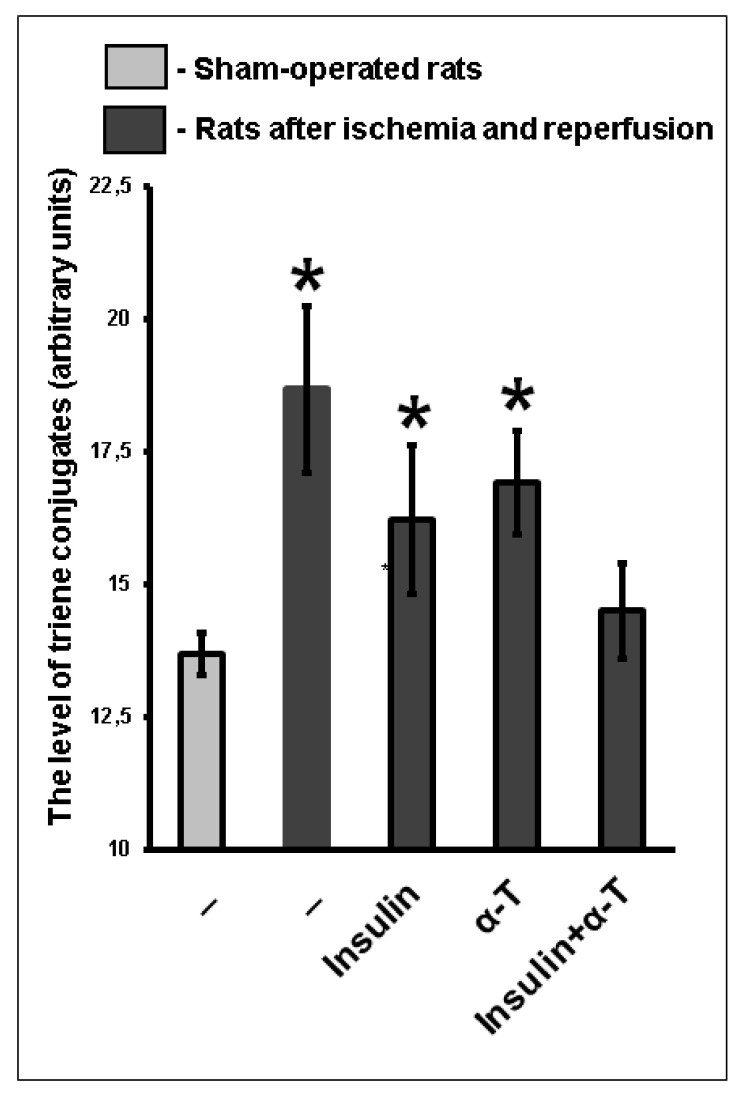
The effect of intranasally administered insulin and orally administered α-T on the level of conjugated trienes in the brain cortex of rats with two-vessel forebrain ischemia followed by reperfusion. The data of 5–7 experiments are presented as the means ± SEM. The two-vessel forebrain ischemia was induced by ligation of carotid arteries for 20 min and hypotension followed by reperfusion for 1 h. Insulin was administered intranasally at a dose of 0.25 IU/rat, while α-T was given orally at a dose of 50 mg/kg. *—The differences are significant according to Student’s *t*-test as compared to control values in the brain cortex of sham-operated rats, *p* < 0.01.

The similar data were obtained when studying the level of conjugated trienes (Figure 10). The level of conjugated trienes was markedly increased (by 30.6%) in ischemic and reperfused rat brain cortex as compared to sham-operated rats (*p* < 0.01). In the brain cortex of ischemic and reperfused rats which received either insulin (intranasally, 0.25 IU/rat) or α-T (orally, 50 mg/kg), the level of conjugated dienes was higher by 24.3% and 27.2% as compared to sham-operated rats (Figure 10). In the brain cortex of rats with ischemic and reperfused forebrain treated with insulin plus α-T, the levels of conjugated trienes did not differ from those in sham-operated rats (Figure 10).

Thus, the combined use of insulin and α-T prevented an increase in the levels of such LPO products as the conjugated dienes and trienes in brain of rats with ischemia/reperfusion. At the same time, the use of insulin or α-T monotherapy was not effective. These data provide evidence that α-T markedly increases the ability of insulin to normalize the metabolic disturbances in brain, which are induced by the activation of free radical reactions.

### 2.10. Additive Activating Effect of the Intranasally Administered Insulin and Orally Administered α-T on Na^+^,K^+^-ATPase Partially Inactivated in Brain Cortex of Rats with Two-Vessel Ischemia and Subsequent Reperfusion

We studied the ability of intranasally administered insulin (0.25 IU/rat) and of orally administered α-T (50 mg/kg) to normalize the Na^+^,K^+^-ATPase activity which is markedly decreased in the brain cortex of rats with two-vessel forebrain ischemia and subsequent reperfusion. The Na^+^,K^+^-ATPase activity was found to be 24.6 ± 1.27 μmol P_i_/mg of protein/h in control sham-operated rats, but it decreased (approximately by 35%) to 15.95 ± 0.82 μmol P_i_/mg of protein/h in ischemic and reperfused rats. Both insulin and α-T administration significantly increased the activity of Na^+^,K^+^-ATPase in the brain cortex of rats with brain ischemia and subsequent reperfusion, their effects were found to be additive. The most pronounced increase of enzyme activity to 23.1 ± 0.83 μmol P_i_/mg of protein/h was reached if both protectors were administered to such rats (insulin intranasally and α-T orally). The increase of the enzyme activity was in this case approximately equal to the sum of the increase caused by insulin and α-T action (Table 2). Thus, when adding the effects of insulin and α-T, acting separately, the increase in the Na^+^,K^+^-ATPase activity will be 7.9 μmol P_i_/mg of protein/h (2.55 + 5.35 μmol P_i_/mg of protein/h). It is in a good agreement with the increase in enzyme activity obtained in experiments with co-administration of both drugs to rats with ischemic/reperfused forebrain injury (7.15 μmol P_i_/mg of protein/h) (Table 2).

Then, by immunoblotting, the levels of α-2 and α-3 subunits of Na^+^,K^+^-ATPase in the cerebral cortex of ischemic and sham-operated rats and the effect of treatment with insulin and α-T on it were investigated. It was shown that in ischemic rats the level of α-2 and α-3 subunits of Na^+^,K^+^-ATPase did not change (Supplementary Material Figures S1 and S2). Insulin, α-T and their combination significantly increased the α-2 subunit level in ischemic/reperfused rats (Supplementary Material Figure S1). In sham-operated rats, only α-T increased the α-2 subunit level (Supplementary Material Figure S1). In the case of the α-3 subunit of Na^+^,K^+^-ATPase, administration of α-T and both drugs significantly increased the level of this subunit in the cerebral cortex of both ischemic and sham-operated rats (Supplementary Material Figure S2).

## 3. Discussion

A decrease of insulin content and insulin signaling in the brain is characteristic of Alzheimer’s and Parkinson’s diseases and diabetes mellitus. The deficit of insulin signaling leads to the impairment of metabolic processes in brain neurons and their function. It is one of the main causes of the disturbances in learning and memory. The most effective way of insulin delivery to the brain is its intranasal administration, in which insulin enters directly to the brain, bypassing the blood-brain barrier [11,12]. In clinical trials, the intranasal administration of insulin to patients with Alzheimer’s and other neurodegenerative diseases was shown to improve the cognitive functions (see, for example, [15,16,17,18,19]). Meanwhile, in the course of these trials, the first evidence of the development of central insulin resistance appeared. The long-acting insulin analogue Detemir (21 days, 40 IU/patient/day) significantly improved memory in patients with Alzheimer’s disease and moderate cognitive deficits [15], but its improving effect with prolonged use (2–4 months) at the same daily dose disappeared [16]. These data suggest that long-acting insulin Detemir induces the chronic brain hyperinsulinemia, which leads to the insulin resistance of brain cells. Taking into account the possibility of the development of central insulin resistance as a result of prolonged intranasal administration of high-dose insulin, it is important to identify biologically active substances that can enhance the protective effect of insulin and increase its effectiveness at lower doses used for treatment. This can help to prevent a decrease in the insulin sensitivity of neurons with prolonged intranasal administration of this hormone to patients.

As far as the clinical trials of α-T or vitamin E administration to patients are concerned, the data obtained are to a certain extent contradictory (see, for example, [30,31,40]). According to the data obtained by Dysken and co-authors [30], in the trials performed from 2007 to 2012, it was shown that prolonged (more than for two years) administration of vitamin E at a daily dose of 2000 IU to patients with mild to moderate Alzheimer’s disease resulted in slower decline in cognitive functions as compared to placebo group. α-T administration improved the activities of daily living and decreased the caregiver burden [30]. The treatment with α-T of the patients with Alzheimer’s disease having moderately severe impairment slowed the progression of the disease [40]. In this case, the clinical trials also took place for 2 years, and the patients received 2000 IU of α-T daily. At the same time, in this work the beneficial effect of α-T administration for long time to patients with mild cognitive impairment was not revealed [40]. In the Cochrane Review of the clinical trials performed before 2000 year [31], no evidence was revealed that vitamin E or α-T improved cognitive function in patients with mild cognitive impairment or dementia, characteristic to Alzheimer’s disease. Meanwhile, the authors indicated that there was moderate quality evidence from a single study that α-T administration could slow functional decline in patients with Alzheimer’s disease. Vitamin E or α-T was not associated with an increased risk of serious adverse events or mortality in various trials [30,31,40]. If patients with Alzheimer’s disease received every day a mixture of folate, α-T, vitamin B12, S-adenosyl methionine, *N*-acetylcysteine and acetyl-l-carnitine, it prevented the decline of their cognitive performance for a period over 12 months and improved the behavioral and psychological symptoms of dementia [41]. All these data suggest that α-T treatment of humans appears to be safe and may have a positive effect on patients with neurodegenerative diseases.

We have shown that pre-incubation of brain cortex neurons in culture with 1 μM insulin and 50 μM α-T prior to application of hydrogen peroxide markedly increased the viability of these cells (Figure 1). The protective effect of the combination of these drugs was additive, it was significantly higher than the effect of each drug alone (*p* < 0.01). We used long (18 h) pre-incubation with α-T, as in this case its neuroprotective effect is due to modulation of signaling pathways and not to its scavenging effect as in the case of short pre-incubation of neurons with this antioxidant [39,42]. α-T was found to enhance the antiapoptotic effect of insulin as well (Figure 2).

Pre-incubation with either insulin or α-T was found to decrease the formation of ROS in brain cortical neurons induced by hydrogen peroxide. The effect of insulin plus α-T was more pronounced (Figure 3) than the effect of each of them, and the difference was significant (*p* < 0.05). Hydrogen peroxide-induced oxidative stress in brain cortical neuron resulted in the pronounced decrease in mitochondrial membrane potential. The pre-incubation of the neurons with insulin plus α-T or with either insulin or α-T alone led to the significant increase in ∆ψ(m), the effect of insulin plus α-T being more pronounced (Figure 7) than the effect of each compound used separately (*p* < 0.01).

The enhancement of the protective effect of insulin by α-T was not shown before our study. So, the mechanism of this effect is not known yet. In order to obtain the first data for understanding of this mechanism we studied the effect of insulin, α-T and their combination on the activity of Akt-kinase, GSK-3beta and ERK1/2. We estimated the ratio pAkt (Ser^473^)/Akt to show the activation of Akt. Insulin activated Akt-kinase both in control cortical neurons and in the neurons 15 min and 6 h after application of hydrogen peroxide (Figure 4). Meanwhile, we did not reveal the significant activating effect of α-T on Akt at the same time points. In our previous studies we have found that not only insulin [43], but α-T as well is able to activate Akt-kinase in brain cortical neurons under oxidative stress conditions [39], but the effect of these two drugs was detected at different time points after pro-oxidant addition [39,43]. In the present work it was shown that Akt activity in brain cortical neurons 6 h after the application of pro-oxidant is significantly higher in the case of application of insulin plus α-T (Figure 4) as compared to application of insulin or α-T alone (*p* < 0.01 by paired Student’s *t*-test in both cases).

Activation of Akt has a protective effect as it increases the synthesis of the antiapoptotic protein Bcl-2 and the ratio Bcl-2/Bax, and inactivates kinase GSK-3beta, phosphorylating it at the Ser^9^ residue. The activation of GSK-3beta leads to the decrease in mitochondrial membrane potential, to the opening of mitochondrial permeability transition pores (mPTP) and to the increase in ROS formation in various cells, including neurons [44,45,46]. The inhibition of GSK-3beta improves the mitochondrial function. In the present study, we have shown that pre-incubation of brain cortical neurons with insulin significantly increased the phosphorylation of GSK-3beta at the Ser^9^ in control neurons and in the neurons 15 min and 6 h after the application of hydrogen peroxide to the cells (Figure 5). It means that insulin inactivated GSK-3beta. We have not revealed the effect of α-T on the phosphorylation of GSK-3beta in control cells and 15 min after addition of prooxidant. At the same time in neurons 6 h after the application of hydrogen peroxide, the use of α-T in the combination with insulin increased the phosphorylation of GSK-3beta and hence enhanced the insulin-induced inactivation of this enzyme (Figure 5). The difference between the effect of insulin plus α-T and the effects of each of them was significant (*p* < 0.01 by paired Student’s *t*-test). The ability of α-T to enhance the Akt activation and GSK-3beta inactivation, induced by insulin, explains to a certain extent its ability to increase the protective effect of insulin on brain cortical neurons observed in our study.

The modulation of ERK1/2 activity by insulin and α-T may also contribute to the ability of these drugs to enhance the protective effect of each other on the nerve cells. Previously, short activation of ERK1/2 by various compounds was shown to increase the viability of neurons [34,35,36,37], but long-term activation of ERK1/2 promoted nerve cells death [35,38]. In ischemic brain, the persistent activation of ERK1/2 occurs, and the ERK1/2 inhibitors were found to improve the brain metabolism and functional state of animals [47,48,49,50]. In the present study, we showed that in cultured brain cortical neurons, 6 h after the addition of hydrogen peroxide the ERK1/2 activity was increased. Pre-incubation of the neurons with insulin or α-T decreased the activity of ERK1/2, and the combination of insulin and α-T resulted in a more pronounced decrease in ERK1/2 activity, normalizing it in cortical neurons.

Thus, according to the data obtained in the present study, in brain cortical neurons, α-T is able to increase the insulin-induced activation of Akt-kinase and to intensify the insulin-induced inactivation of GSK-3beta and ERK1/2 at late stages of oxidative stress, thereby increasing the neuroprotective effects of insulin. Most probably, the ability of insulin and α-T to increase the neuroprotective effects of each other depends on modulation of other signaling pathways and protein kinases as well, which requires further investigations.

One of the main causes of neuronal damage and death during brain ischemia and subsequent reperfusion is activation of free radical reactions. An important task was to find out whether insulin and α-T are capable of enhancing each other’s effects on metabolic abnormalities caused by the activation of free radical reactions, which are characteristic of two-vessel forebrain ischemia with hypotension and subsequent reperfusion.

It may be emphasized that in order to reveal the additive effect of insulin and α-T, we applied much lower doses of these drugs than are usually used when studying their protective effects in vivo. Effects of insulin administered intranasally to rats are usually studied using its dose of 0.5 IU/rat [51,52]. As far as α-T is concerned, administration of 125 or even 250 mg/kg of this compound to rats orally is used frequently when studying the protection elicited by a single or multiple administration of this drug [53,54]. In our experiments, we administered to rats once 0.25 IU of insulin intranasally and twice 50 mg/kg of α-T orally.

Levels of LPO products such as Schiff bases increased markedly in the brain cortex of rats with two-vessel forebrain ischemia/reperfusion injury. Both intranasal administration of insulin (0.25 IU/rat) and oral administration of α-T (50 mg/kg) to ischemic rats significantly diminished the accumulation of Schiff bases in the brain cortex. The most pronounced diminution of Schiff bases took place at co-administration of these drugs. In this case, the decrease of Schiff bases content was more pronounced as compared to administration of either insulin, or α-T alone, the difference was significant (*p* < 0.01) (Figure 8).

The content of conjugated dienes and trienes also increased markedly in the brain cortex of rats subjected to two-vessel ischemia, hypotension and subsequent reperfusion. Neither intranasally administered insulin, nor orally administered α-T could normalize the content of conjugated dienes or trienes in ischemic rats, as their level remained significantly higher as compared to their level in sham-operated rats. At the same time, co-administration of insulin and α-T led to normalization of the levels of conjugated dienes and trienes in the brain cortex of rats with forebrain ischemia, they became practically equal to control values in the brain cortex of sham-operated rats, the difference was quite small and not significant (Figure 9 and Figure 10). Thus, insulin and α-T enhance the restoration effect of each other on brain metabolic processes under the conditions of activation of free radical reactions, and their combined use more effectively restored redox status of ischemic brain than monotherapy with these drugs.

The enzyme Na^+^,K^+^-ATPase is an important component of all animal cells, and its main function is the maintenance of resting potential of the cells and the regulation of cell volume. In the neurons, the difference in the concentrations of sodium and potassium ions in the cells and in the extracellular space, is a result of Na^+^,K^+^-ATPase activity and is used for propagation of the nerve impulses [55,56]. The decrease in the activity of Na^+^,K^+^-ATPase may be considered as an integral indicator of metabolic and functional impairments in the brain. The normalization of the activity of this enzyme may be an evidence of improvement of brain metabolism and functions. The decrease of Na^+^,K^+^-ATPase activity in ischemic and reperfused brain cortex may be a result of oxidative inactivation of this enzyme due to the decrease of the reduced SH groups and of the inhibitory effect of such LPO products as 4-hydroxynonenal [57,58,59]. The antioxidant effect of α-T and insulin and their ability to reduce accumulation of LPO products contribute to their normalizing effect on Na^+^,K^+^-ATPase activity in the brain cortex of rats with two-vessel forebrain ischemia and subsequent reperfusion.

As we have shown, in the brain cortex of ischemic rats, the Na^+^,K^+^-ATPase activity was decreased significantly as compared to sham-operated rats, and the treatment with insulin or α-T led to an increase in the enzyme activity (*p* < 0.05 and *p* < 0.01, respectively). The co-administration of insulin and α-T used at relatively low doses in the in vivo experiments appears to be effective, and their action was shown to be additive. These drugs enhance the ability of each other to reduce the ROS-induced metabolic disturbances and thereby prevent neurodegenerative processes in the ischemic and reperfused brain.

## 4. Materials and Methods

### 4.1. Materials

Poly-D-lysine, sodium dodecyl sulfate, 3-(4,5-dimethylthiazol-2-thiazolyl)-2,5-diphenyl-2*H*-tetrazolium bromide (MTT), 2′,7′-dichlorofluorescein diacetate, hydrogen peroxide, NADH and insulin were obtained from Sigma-Aldrich (St. Louis, MO, USA). The neurobasal medium, B27 Supplement, B27 supplement without insulin, penicillin/streptomycin solution and glutamax were obtained from Gibco (Paisley, UK). Hanks buffered salt solution and trypsin/versen (1:1) solution were purchased from the Biolot Company (Saint-Petersburg, Russia), and dimethylformamide was obtained from Vecton (Saint-Petersburg, Russia). The primary and secondary antibodies and other reagents used for immunoblotting are described in Section 4.6.

### 4.2. Brain Cortical Neurons in Culture

Immature brain cortical neurons were isolated from embryonic day 17–18 Wistar rat fetuses as previously described [60]. All procedures of using animals were performed in accordance with the European Community Council Directive 1986 (2010/63/EEC) and “Guide for the care and use of laboratory animals”. They were approved by the Bioethics committee of the Institute of Evolutionary Physiology and Biochemistry of Russian Ac. Sci. In order to prepare primary cultures of immature brain cortical neurons the isolated cells were seeded on poly-D-lysine-coated 24-well and 12-well plates at a density of 5 × 10^5^ and 1 × 10^6^ cells per well, respectively. Cells were cultured in neurobasal medium containing 2% of B27 supplement, 2 mM glutamax, 100 U/mL of penicillin and 100 μg/mL of streptomycin (growth medium). Every three days half of the culture medium was replaced. The beginning of the experiments took place on the 5th–6th day in vitro of neuron cultivation in the growth medium. Before the start of the experimental work the growth medium was replaced by neurobasal medium containing 1% B27 supplement without insulin, the neurons were left in it overnight.

### 4.3. Determination of the Viability of Brain Cortical Neurons by MTT Method and of Activation of Caspoase-3 Measuring the Level of Its 17–19 kDa Fragment

Brain cortical neurons were preincubated with 50 μM α-T for 18 h or with 1 μM insulin for 1 h or with both protectors or without them in the neurobasal medium containing 1% B27 without insulin. Then the cells were exposed to 100 μM hydrogen peroxide for 6 h. The viability of brain cortical neurons in culture was evaluated by means of colorimetric 3-(4,5-dimethylthiazol-2-yl)-2,5-diphenyltetrazolium bromide (MTT) method. The MTT assay which is based on the ability of mitochondria of viable cells to reduce MTT to purple-colored MTT-formazan was conducted as previously described [61]. A microplate reader “Anthos” was used to measure the absorbance of MTT-formazan at 575 nm. The extinction of the samples was expressed as percent of the extinction of control samples, which was taken for 100%.

The caspase-3 activity in brain cortical neurons and hence the apoptotic death of the cells was estimated measuring the level of cleaved caspase-3, that is the level of its 17–19 kDa fragment in control neurons and in neurons 15 min and 6 h after the initiation of the oxidative stress in the cells by application of 100 μM hydrogen peroxide. The level of this fragment shows the degree of cleavage and activation of caspase-3. It was measured using immunoblotting technique.

### 4.4. Determination of ROS Formation in Brain Cortical Neurons

Brain cortical neurons were preincubated with 50 μM α-T for 18 h or with 1 μM insulin for 1 h or with both protectors or without them in the neurobasal medium containing 1% of B27 Supplement without insulin. Then the cells were switched to Hanks’ balanced salt solution (HBSS) to start loading of 10 μM dye 2′,7′-dichlorofluorescein diacetate for 40 min at 37 °C in the dark. After washing twice with HBSS, the neurons were exposed to 100 μM hydrogen peroxide for 1 h. The fluorescence of the reaction product of 2′,7′-dichlorofluorescein with ROS was determined using a Fluoroscan Ascent FL (Thermo Fisher Scientific, Helsinki, Finland) fluorimeter for plates (excitation at λ = 485 nm, emission measured at λ = 538 nm). The ROS levels in the samples were determined using arbitrary units.

### 4.5. Evaluation of Insulin, α-T and Hydrogen Peroxide Effects on Akt, GSK-3beta and ERK1/2 Activities and Expression of These Protein Kinases and of Cleaved Caspase-3 Using Western Blot Analysis

After pre-incubation with 50 μM α-T for 18 h or 1 μM insulin for 1 h or with both protectors or without them hydrogen peroxide was added to the samples to the final concentration of 100 μM for 6 h. The cortical neurons were washed twice with ice-cold phosphate buffer solution (PBS). Then they were harvested in 60 μL of lysis buffer. The lysis of cortical neurons and determination of protein concentrations were performed as previously described [39].

The equal amounts of lysates (20–25 μg of protein) were loaded into each lane on 10% sodium dodecyl sulfate polyacrylamide gel. The electrophoresis of the samples was made at constant voltage. Afterwards, the proteins were transferred to 0.45 μm Protran nitrocellulose membranes (Amersham, GE Healthcare, Little Chalfont, Buckinghamshire, UK). The nonspecific binding sites of the membranes were blocked as it was previously described [39]. Monoclonal antibodies specific for pAkt (Ser^473^) (1:1000, #4058, Cell Signaling Technology, Danvers, MA, USA), for pGSK-3beta (Ser^9^) (1:1000, #9322, Cell Signaling Technology), for pERK1 (pThr^202^/pTyr^204^) and pERK2 (pThr^185^/pTyr^187^) (1:2000, #E7028, Sigma-Aldrich), and for cleaved caspase-3 (Asp175) (1:1000, #9664, Cell Signaling Technology) were used to probe the blots overnight at +4 °C. The specific antibodies to total Akt (1:1000, #4691, Cell Signaling Technology), ERK1/2 (1:1000, #9102, Cell Signaling Technology) and GSK-3beta (1:1000, #9315, Cell Signaling Technology) were used to determine the level of these enzymes and the possible changes in their expression. After incubation with primary antibodies the blots were washed three times with 0.1% Tween 20 in Tris-buffered saline (50 mM Tris (pH 7,5), 150 mM NaCl) and incubated with either anti-mouse (#7076) or anti-rabbit (#7074) HRP-IgG secondary antibody (Cell Signaling Technology) diluted in 5% nonfat milk with TBST buffer for 1 h at room temperature. The ratios pAkt (Ser^473^)/Akt, pERK1/2/Total ERK1/2, pGSK-3beta (Ser^9^)/GSK-3beta were determined and taken as 1.0 in control cells. In order to normalize the data, membranes after stripping were re-probed for α-tubulin (1:2000, #T6074, Sigma-Aldrich). To check the changes of the expression of total ERK1/2, Akt and GSK-3beta, their ratio to α-tubulin was determined and taken as 1.0 in control neurons. The stripping procedure was previously described [39]. The ratios of cleaved caspase-3 (17–19 kDa fragment)/α-tubulin were also determined. These ratio in control cells was taken as 1.00. Blots were developed with an Novex ECL HRP enhanced chemiluminescence detection reagent Kit (Invitrogen, Waltham, MA, USA). The films were scanned on a CanoScan 8800F scanner (Canon, Tokyo, Japan). Bio7 was used to quantify the optical densities of the positive bands.

### 4.6. Determination of the Effects of Hydrogen Peroxide Application and of Pre-Incubation with Insulin and α-Tocopherol on Mitochondrial Membrane Potentials in Brain Cortical Neurons in Culture

Evaluation of mitochondrial membrane potentials (∆ψ(m)) in brain cortical neurons was performed by the flow cytometry method using the fluorescent dye tetramethylrhodamine (TMRM) [62]. Prior to the beginning of the experiments the growth medium was changed to neurobasal medium containing 1% of B27 supplement without insulin and brain cortical neurons were incubated in it for 6 h. Then the neurons were pre-incubated with insulin and/or α-T for 20 h in this medium, afterward hydrogen peroxide was added to the samples to the final concentration of 100 μM and the neurons were incubated for 1 h. The cells were taken off the wells by trypsin–versen (1:1) and washed by HBSS. The residue was resuspended in HBSS containing 100 nM TMRM. Then the incubation with it for 30–40 min at 37 °C in the dark was performed. The samples were analyzed by flow-cytometry method using an Epics XL cytometer (Beckman Coulter, Brea, CA, USA. Fluorescence was measured at 575 nm in an F2 channel. At least 10,000 events were recorded in each sample. The intensity of fluorescence was shown on logarithmic scale, it was expressed in arbitrary units as the mean intensity of fluorescence. The program WinMDI, version 2.9 was used in order to analyze the cytofluorometric data.

### 4.7. Two-Vessel Forebrain Ischemia in Wistar Rats

The experiments were performed using Wistar rats kept in standard vivarium conditions and weighing 270–330 g. All procedures of using animals were performed in accordance with the European Community Council Directive 1986 (2010/63/EEC) and “Guide for the care and use of laboratory animals”. They were approved by the Bioethics committee of the Institute of Evolutionary Physiology and Biochemistry of Russian Ac. Sci. Chloral hydrate (400 mg/kg body weight) was used for anesthesia. Two-vessel forebrain ischemia was induced by 20-min occlusion of carotid arteries in combination with hypotension. Hypotension was induced by blood sampling until reaching arterial pressure of 50 mm Hg as it was described previously [63]. Before blood sampling rats were injected with 0.2 mL 0.9% NaCl containing 10 IU of heparin, then blood was taken in the same syringe. Upon cessation of 20-min occlusion, the carotid arteries were unclamped and heparinized blood withdrawn at the ischemic stage was returned back to the cerebral circulation. The brain was reperfused for 1 h. The data obtained for sham-operated rats were used as controls. Intranasal administration of insulin was performed 1 h before occlusion of carotid arteries by putting 10 μL of citrate buffer solution containing insulin into each nostril. Citrate buffer was prepared from equal volumes of 100 mM citric acid and 100 mM sodium citrate, pH 4.4. The solution of citrate buffer contained 0.5 mg insulin in 1 mL. It corresponds to insulin dose of 0.25 IU in 20 μL of the solution, which was administered to rats intranasally in our experiments. α-T was given to rats per-orally (50 mg per kg of rat body weight in the evening a day before operation and 1 h before the beginning of operation.

### 4.8. Determination of Lipid Peroxidation Products, Na^+^,K^+^-ATPase Activity and Na^+^,K^+^-ATPase subunits expression in Brain Cortex of Rats after Two-Vessel Ischemia and Reperfusion

Upon cessation of reperfusion, animals were decapitated, with the brain cortex withdrawn thereafter. The level of various lipid peroxidation products was studied in lipid extracts of the rat brain cortex. Lipids were extracted with chloroform-methanol (2:1), as previously described [64]. In order to get rid from nonlipid admixtures the extracts were washed with cooled 0.9% NaCl solution in water (0.2 volumes of the lipid extract) as recommended by Folch and co-authors. Then the surface of the lower phase was washed twice with a “theoretical upper layer”–chloroform:methanol:H_2_O mixture (3:48:47). Then the lipid extract was evaporated and dissolved in chloroform. The fluorescent intensity of Schiff bases was measured on a RF 1501 spectrofluorometer (Shimadzu, Kyoto, Japan) (excitation at λ = 370 nm, emission measured at λ = 450 nm [65]. The results were expressed as relative units (fluorescence units) per 1 mg of lipids. To determine the level of conjugated diene and triene, the evaporated lipid extract was dissolved in a mixture of methanol and hexane (5:1). Ultraviolet absorbance, measured at 232 and 274 nm, was employed to monitor the formation of conjugated dienes and trienes, respectively, and the measurement was performed on spectrophotometer UV-2401 (Shimadzu). The increasing absorption values are an indication that lipid peroxidation is proceeding [66]. The content of conjugated dienes and trienes was expressed in arbitrary units per 1 mg of lipids.

Na^+^,K^+^-ATPase activity was determined in the crude synaptosome fraction (P_2_-fraction) from the rat brain cortex by a coupled reaction in the presence of excess pyruvate kinase, lactate dehydrogenase and phosphoenolpyruvate as reported previously [67]. Na^+^,K^+^-ATPase activity was expressed in μmoles of inorganic phosphate (P_i_) per 1 mg protein for 1 h.

The level of α-2 and α-3 subunit of Na^+^,K^+^-ATPase in the rat brain cortex was measured by means of Western blot analysis. The dissected brain cortex tissues were homogenized in the ratio 1:20 in the lysis buffer. This buffer contained 20 mM Tris-HCl (pH 7.5), 150 mM NaCl, 2 mM EGTA, 2 mM EDTA, 0.5% sodium deoxycholate, 0.5% Triton X-100, 15 mM NaF, 10 mM sodium glycerophosphate, 10 mM sodium pyrophosphate, 1 mM Na_3_VO_4_, 1 mM phenylmethylsulfonyl fluoride (PMSF), 0.02% NaN_3_, and the protease inhibitor cocktail (Roche, Switzerland). The cell fragments and the undamaged cells were separated by centrifugation at 500× *g* for 10 min (4 °C). The protein concentration was measured by the Lowry method with BSA as a standard. Twenty micrograms of protein per sample were run on 9% SDS-polyacrylamide gel. Specific antibodies raised against Sodium Pump subunit α-2 (1:1000, #LS-C368564, LifeSpan BioSciences Inc., Seattle, WA, USA) and Sodium Potassium ATPase α3 subunit (1:1000, #NB300-540, Novus Biologicals, Centennial, CO, USA) were applied for immunostaining in Western Blot Analysis. To normalize the data, the membranes were treated with the antibodies against glyceraldehyde 3-phosphate dehydrogenase (GAPDH) (1:5000, #NB600-502, Novus Biologicals).

### 4.9. Statistical Analysis

Data are presented as mean ± SEM. The statistical significance of the differences between three or more groups of data was assessed by one-way analysis of variance (ANOVA) followed by Tukey’s test for multiple comparisons. Statistical significance of differences between two groups of data was determined using the Student’s *t*-test or Student’s paired *t*-test. Student’s paired *t* test was used in the case of immunoblotting experiments performed on primary cultures of brain cortex neurons. Differences were considered significant at *p* < 0.05.

## 5. Conclusions

Clinical trials show that insulin administered intranasally is promising as a drug for neurodegenerative and, probably. other diseases associated with brain damage. However, long-term systemic or intranasal administration of high-dose insulin can lead to insulin resistance, including the brain insulin resistance. It seems of importance to reveal endogenous or exogenous substances that can enhance the neuroprotective effect of insulin, and these substances may include α-T, the main and most active component of vitamin E. The aim of our work was to study the effectiveness of the insulin plus α-T combination to prevent the death of cultured brain cortical neurons under conditions of oxidative stress and to normalize ROS-induced metabolic dysfunctions in the cerebral cortex of rats with two-vessel forebrain ischemia/reperfusion injury.

In our study, it was for the first time shown that α-T significantly enhanced the protective, antiapoptotic, and antioxidative effects of insulin on cortical neurons under conditions of oxidative stress. The protective and antioxidative effects of these drugs were additive. Pre-incubation of neurons with 1 μM insulin plus 50 μM α-T increased the viability of brain cortical neurons more than pre-incubation with either of these drugs. The pronounced increase of cleaved caspase-3 level (17–19 kDa) and hence of caspase-3 activation was observed 6 h after the beginning of the exposure of neurons to 100 μM hydrogen peroxide. The combined use of insulin and α-T decreased the level of cleaved caspase-3 (and, consequently, the apoptotic death of neurons) to a greater extent than pre-incubation with each of these drugs. In cortical neurons, the antioxidant effect of insulin plus α-T was more pronounced than in the case of the application of insulin or α-T alone.

As the enhancement of neuroprotective effect of insulin by α-T was first shown in our study, the mechanisms of this effect were not known. In order to decipher them, we studied the modulation of the activity of various protein kinases by insulin, α-T and insulin plus α-T in cultured brain cortical neurons under conditions of oxidative stress, induced by hydrogen peroxide. At the later stages of oxidative stress, the combined action of insulin and α-T led to an increase in Akt activity, inactivation of GSK 3beta and normalization of ERK1/2 activity, and these effects were more pronounced than those of either drug.

In rats, forebrain ischemia and subsequent reperfusion increased the content of LPO products and caused oxidative inactivation of Na^+^,K^+^-ATPase in the cerebral cortex. Co-administration of insulin and α-T resulted in a more pronounced normalization of the levels of Schiff bases, conjugated dienes and trienes and the activity of Na^+^,K^+^-ATPase than the administration of one of these drugs. As activation of free radical reactions is one of the main causes of nerve cell damage and death in the ischemic and reperfused brain, these data suggest that α-T is able to enhance the neuroprotective effect of insulin in ischemic conditions followed by reperfusion. Further experiments will show whether the combined use of insulin and α-T is able to prevent effectively neuropathies characteristic of neurodegenerative, ischemic and other brain injuries.

## Figures and Tables

**Table 1:** The effect of pre-incubation with insulin and α-tocopherol (α-T) on mitochondrial membrane potential in brain cortical neurons exposed to hydrogen peroxide.

Sample	Mean ± SEM
Control	71.1 ± 2.88
Control+Insulin	67.5 ± 0.15
Control+ α-T	68.3 ± 0.25
Hydrogen peroxide (HP)	55.9 ± 4.64 ^a^
HP+ Insulin	78.3 ± 0.72 ^b^
HP+ α-T	68.0 ± 0.36 ^b^
HP+Insulin+ α-T	89.0 ± 2.36 ^b,c^

Abbreviations used: α-tocopherol (α-T). Brain cortical neurons in culture were pre-incubated with 1 μM insulin or with 50 μM α-T or with both protectors or without them for 20 h and then exposed for 1 h to 100 μM hydrogen peroxide. The data are given as the means ± SEM of 4–5 determinations. The mitochondrial membrane potential was measured using TMRM on a Beckman Coulter Epics XL flow cytometer. The data obtained were expressed in arbitrary units. The differences are significant according to Student’s *t*-test: ^a^—as compared to control values, *p* < 0.05, ^b^—as compared to the effect of hydrogen peroxide alone, *p* < 0.02, ^c^—as compared to the effect of hydrogen peroxide+insulin and hydrogen peroxide+ α-T, *p* < 0.01.

**Table 2:** The effect of intranasally administered insulin and per-orally administered α-T on Na^+^,K^+^-ATPase activity in brain cortex of rats with two-vessel ischemia followed by reperfusion (μmol P_i_/mg of protein/h).

Sham-Operated or Ischemic and Reperfused Rats	Administration to Rats	Na^+^,K^+^-ATPase Activity (μmol P_i_/mg of Protein/h)
Sham-operated rats	-	24.6 ± 1.27
Sham-operated rats	0.25 IU insulin	22.7 ± 0.48
Sham-operated rats	50 mg α-T per kg	24.2 ± 0.51
Sham-operated rats	0.25 IU insulin and 50 mg α-T per kg	24.4 ± 0.74
Ischemic and reperfused rats	-	15.95 ± 0.82 ^a^
Ischemic and reperfused rats	0.25 IU insulin	18.5 ± 0.82 ^a,b^
Ischemic and reperfused rats	50 mg α-T per kg	21.3 ± 0.83 ^c^
Ischemic and reperfused rats	0.25 IU insulin and 50 mg α-T per kg	23.1 ± 0.83 ^c,d^

The data of 6–8 experiments are given as the means ± SEM. The two-vessel ischemia was induced by ligation of carotid arteries for 20 min and hypotension followed by reperfusion for 1 h. Insulin was administered intranasally in a dose of 0.25 IU, while α-T was given per-orally in a dose of 50 mg per kg of rat body weight. The differences are significant according to Students’ *t*-test as compared: ^a^—with control values in sham-operated rats, *p* < 0.01, ^b,c^ with the effect of ischemia and reperfusion, ^b^ *p* < 0.05, ^c^ *p* < 0.001, ^d^ with the effect of insulin alone, *p* < 0.01.

## Data Availability

Data is contained within the article or supplementary material.

## Supplementary Materials

The following are available online at https://www.mdpi.com/article/10.3390/ijms222111768/s1.

## Abbreviations

α-T	α-Tocopherol
Akt	Protein kinase B
ERK1/2	Extracellular signal-regulated protein kinase
GSK-3beta	Glycogen synthase kinase-3beta
IGF-1	Insulin-like growth factor-1
LPO	Lipid peroxidation
MTT	3-(4,5-Dimethyl-2-thiazolyl)-2,5-diphenyl-2*H*-tetrazolium bromide
ROS	Reactive oxygen species
TMRM	Tetramethylrhodamine methyl ester
