# Supplementary material for: Insulin and α-Tocopherol Enhance the Protective Effect of Each Other on Brain Cortical Neurons under Oxidative Stress Conditions and in Rat Two-Vessel Forebrain Ischemia/Reperfusion Injury
Source: Int J Mol Sci. 2021 Oct 29;22(21):11768. doi: 10.3390/ijms222111768 (PMC8584186; doi:10.3390/ijms222111768)
Supplement: Supplementary file 1 [file ijms-22-11768-s001.zip › ijms-1420101-supplementary materials.pdf]

International Journal of *Molecular Sciences*

MDPI

Supplementary materials

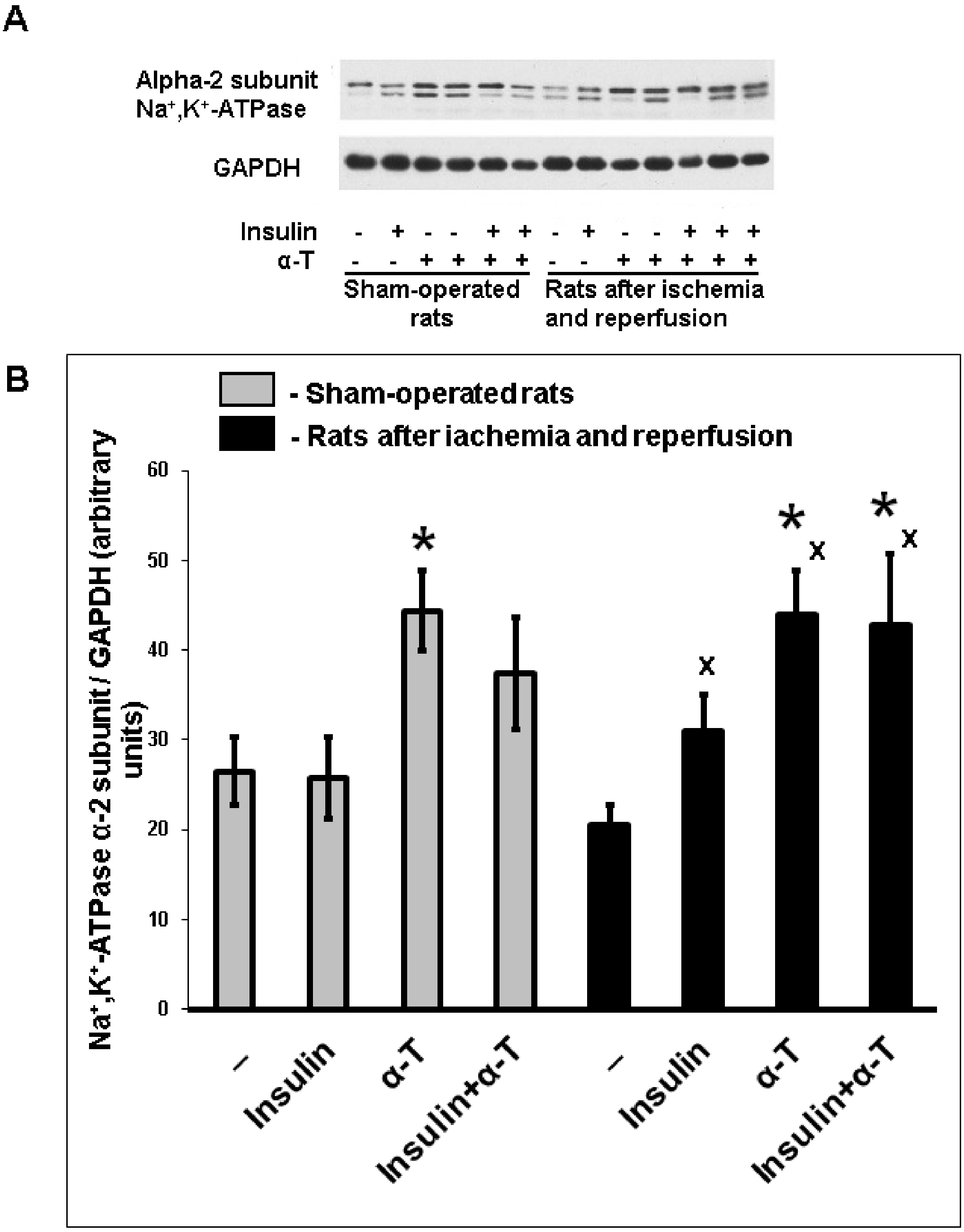


**Figure S1.** The effect of intranasally administered insulin and per-orally administered $\alpha$-T on the level of $\alpha$-2 subunit of $Na^+,K^+$-ATPase in brain cortex of rats with two-vessel ischemia followed by reperfusion. A—immunoblots showing the level of $\alpha$-2 subunit of $Na^+,K^+$-ATPase in brain cortex of rats with two-vessel ischemia followed by reperfusion. B—the data of 5–7 experiments are presented as means ± SEM. The two-vessel ischemia was induced by ligation of carotid arteries for 20 minutes and hypotension followed by reperfusion for 1 h. Insulin was administered intranasally in a dose of 0.25 IU, while $\alpha$-T was given twice per-orally in a dose of 50 mg per kg of rat body weight. The differences are significant according to Student's *t*-test as compared with: *—control values in sham-operated rats, $p > 0.05$, x—the effect of ischemia and reperfusion, $p < 0.05$.

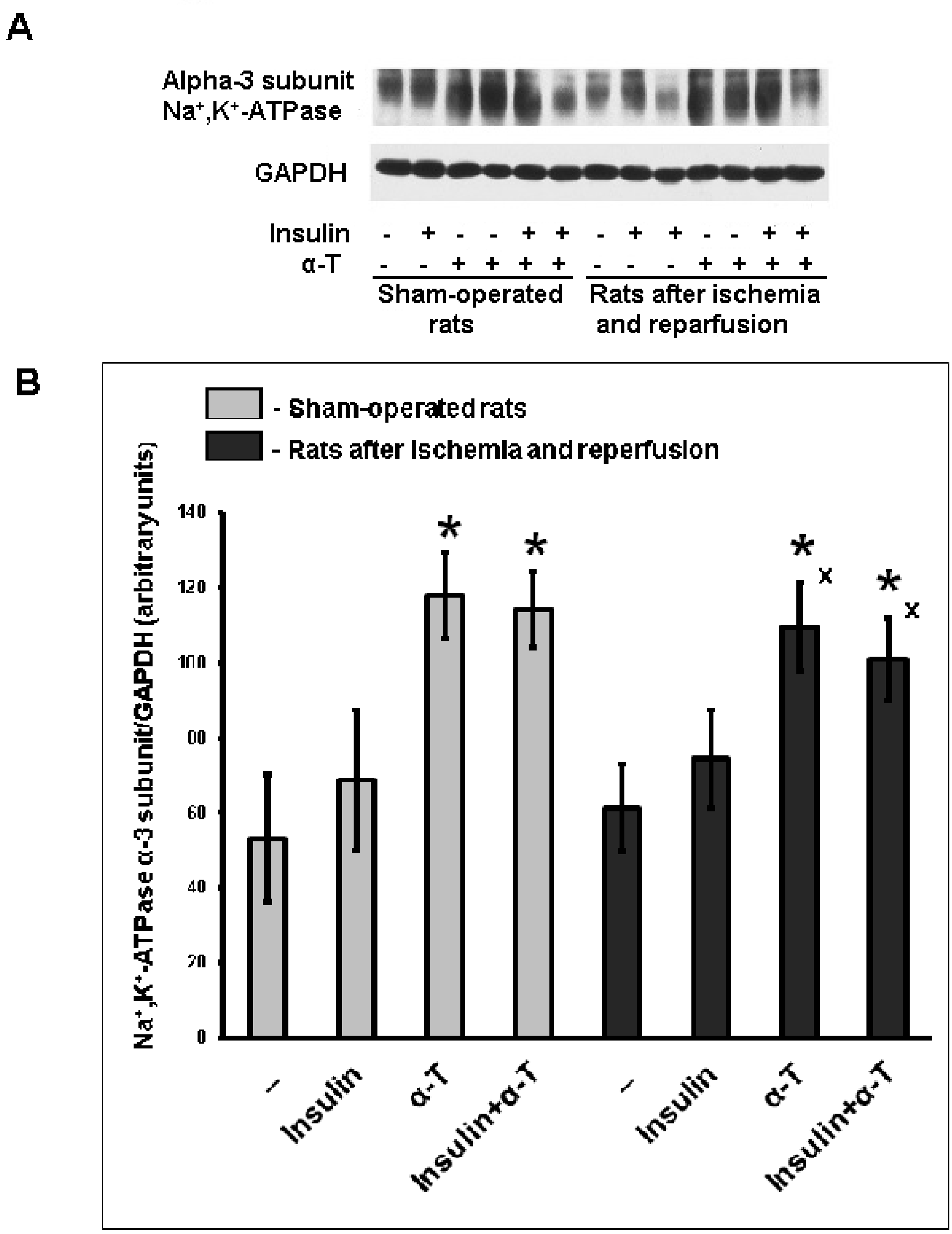


**Figure S2.** The effect of intranasally administered insulin and per-orally administered $\alpha$-T on the level of $\alpha$-3 subunit of $Na^+,K^+$-ATPase in brain cortex of rats with two-vessel ischemia followed by reperfusion. A—immunoblots showing the level of $\alpha$-3 subunit of $Na^+,K^+$-ATPase in brain cortex of rats with two-vessel ischemia followed by reperfusion. B—the data of 5–7 experiments are presented as means ± SEM. The two-vessel ischemia was induced by ligation of carotid arteries for 20 minutes and hypotension followed by reperfusion for 1 h. Insulin was administered intranasally in a dose of 0.25 IU, while $\alpha$-T was given twice per-orally in a dose of 50 mg per kg of rat body weight. The differences are significant according to Student's *t*-test as compared with: *—control values in sham-operated rats, $p > 0.05$, x—the effect of ischemia and reperfusion, $p < 0.05$.
